# Supplementary material for: Detecting heterogeneity in single-cell RNA-Seq data by non-negative matrix factorization
Source: PeerJ. 2017 Jan 19;5:e2888. doi: 10.7717/peerj.2888 (PMC5251935; doi:10.7717/peerj.2888)
Supplement: Figure S1 — (A) PCA plot showing wrongly clusters samples by K-means. (B–D) Dendrogram showing the clustering results of hierarchical clustering, the red dashed lines are the binary decisions from the roots. [file peerj-05-2888-s001.pdf]

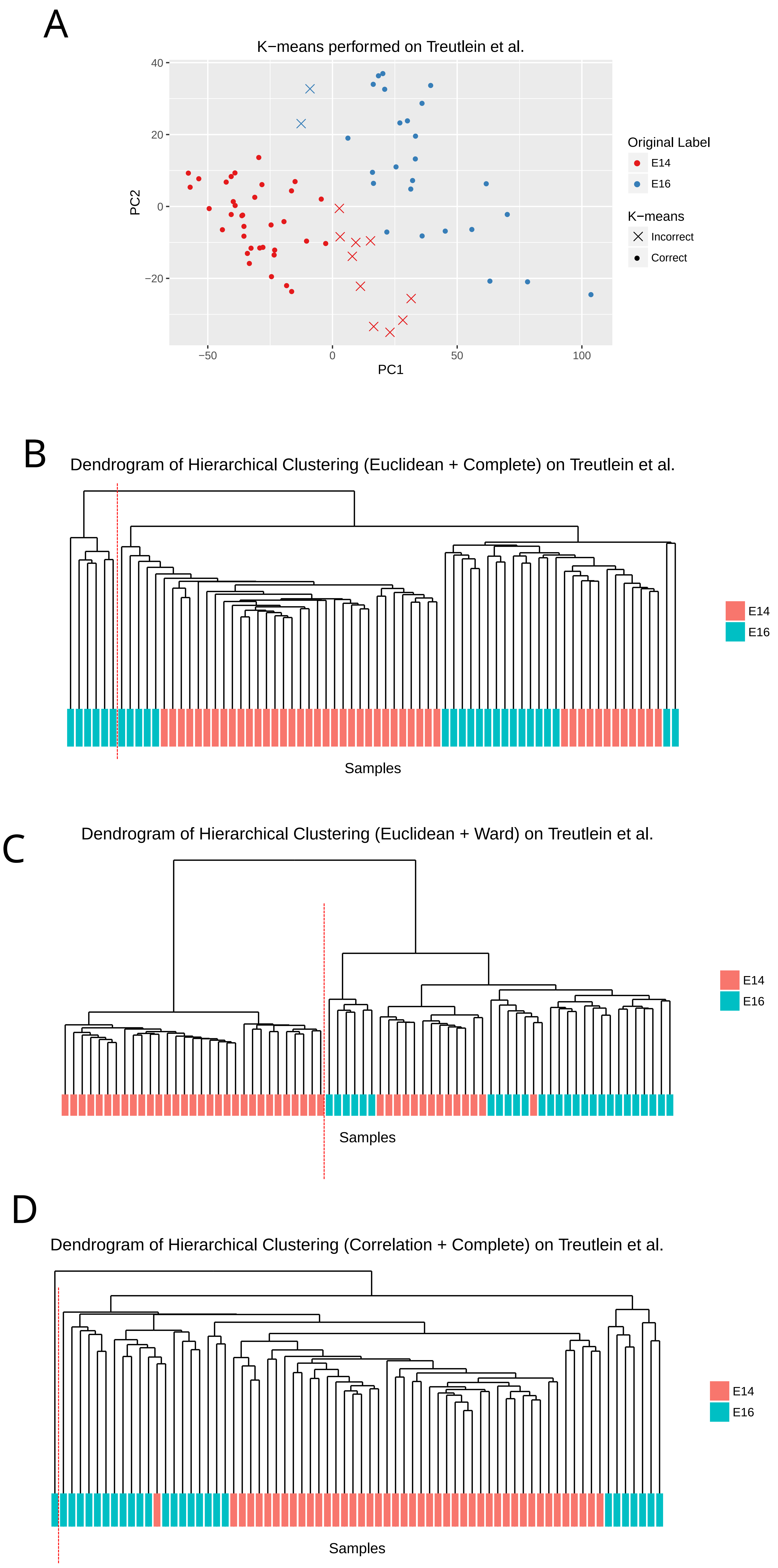
A
K−means performed on Treutlein et al.
PC2
PC1
40
20
0
−20
−50
0
50
100
Original Label
E14
E16
K−means
Incorrect
Correct
B
Dendrogram of Hierarchical Clustering (Euclidean + Complete) on Treutlein et al.
E14
E16
Samples
C
Dendrogram of Hierarchical Clustering (Euclidean + Ward) on Treutlein et al.
E14
E16
Samples
D
Dendrogram of Hierarchical Clustering (Correlation + Complete) on Treutlein et al.
E14
E16
Samples
