# Supplementary figures and images for: Detecting heterogeneity in single-cell RNA-Seq data by non-negative matrix factorization
Source: PeerJ. 2017 Jan 19;5:e2888. doi: 10.7717/peerj.2888 (PMC5251935; doi:10.7717/peerj.2888)

A

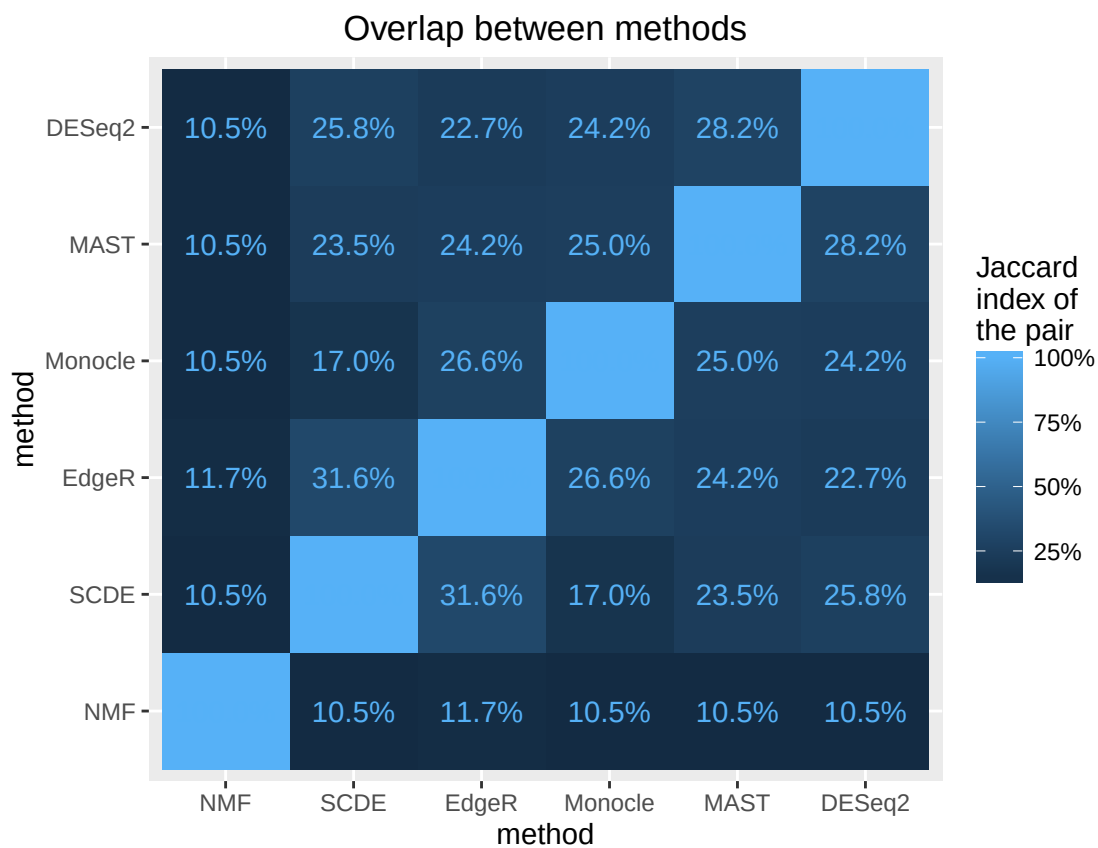

B

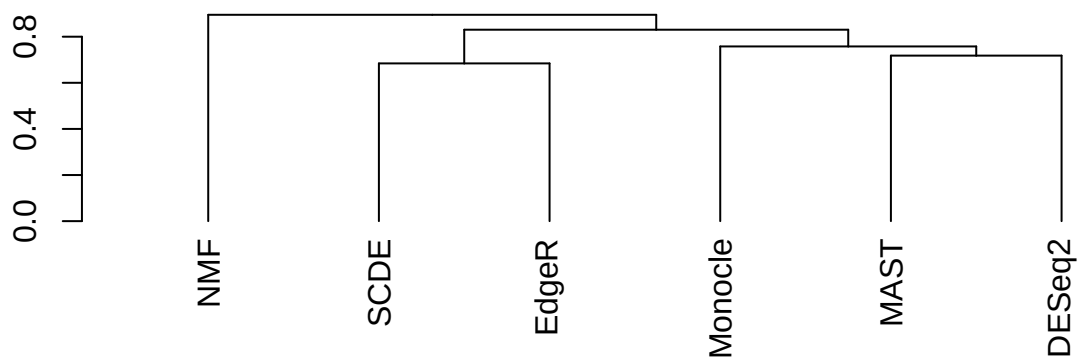

Supplement: Figure S2 — (A) heatmap with each square marking the Jaccard index of the pair. (B) The dendrogram showing the hierarchical clustering results using the distance measured by one minus the Jaccard indices. [file peerj-05-2888-s002.pdf]

A

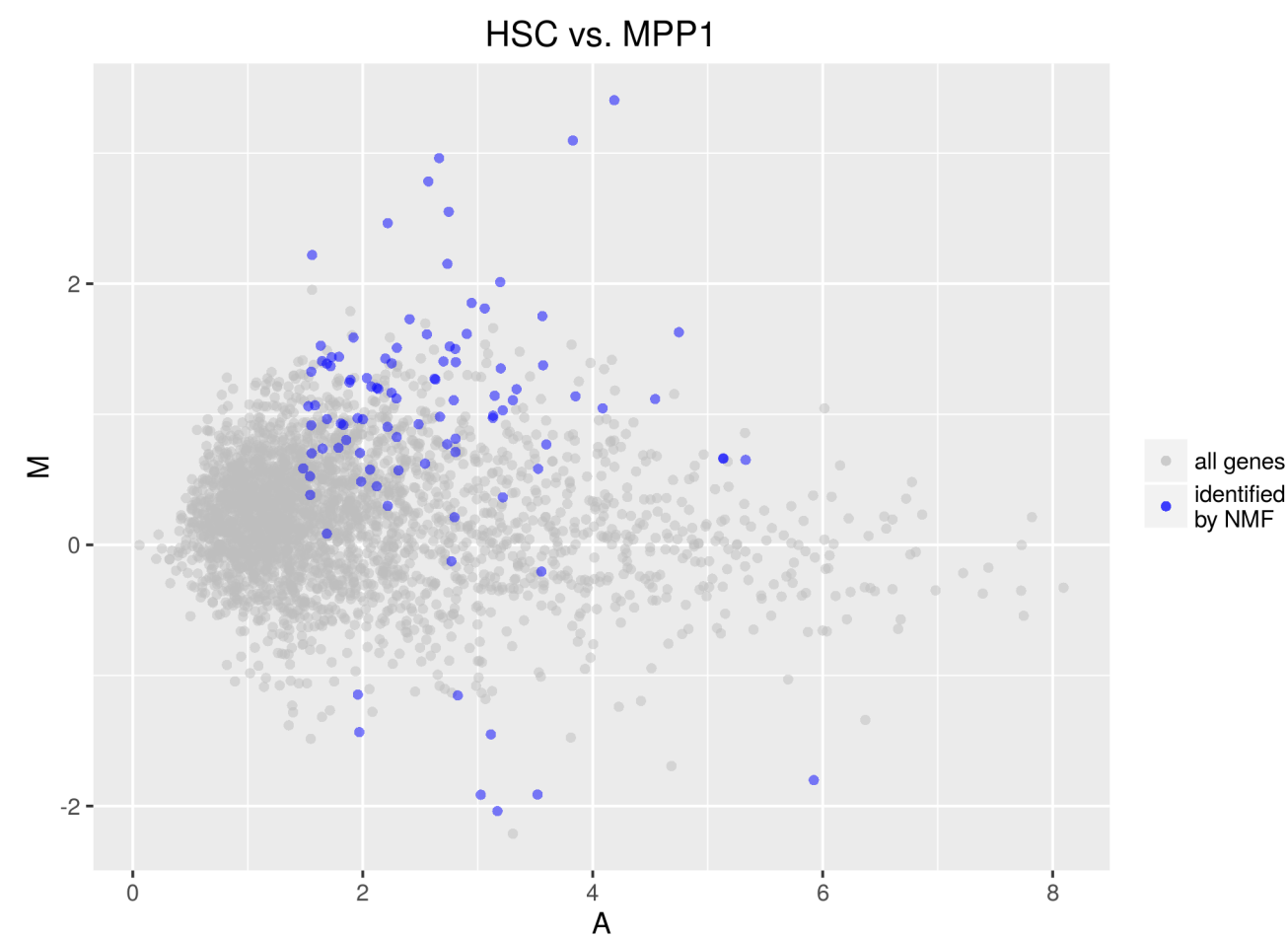

B

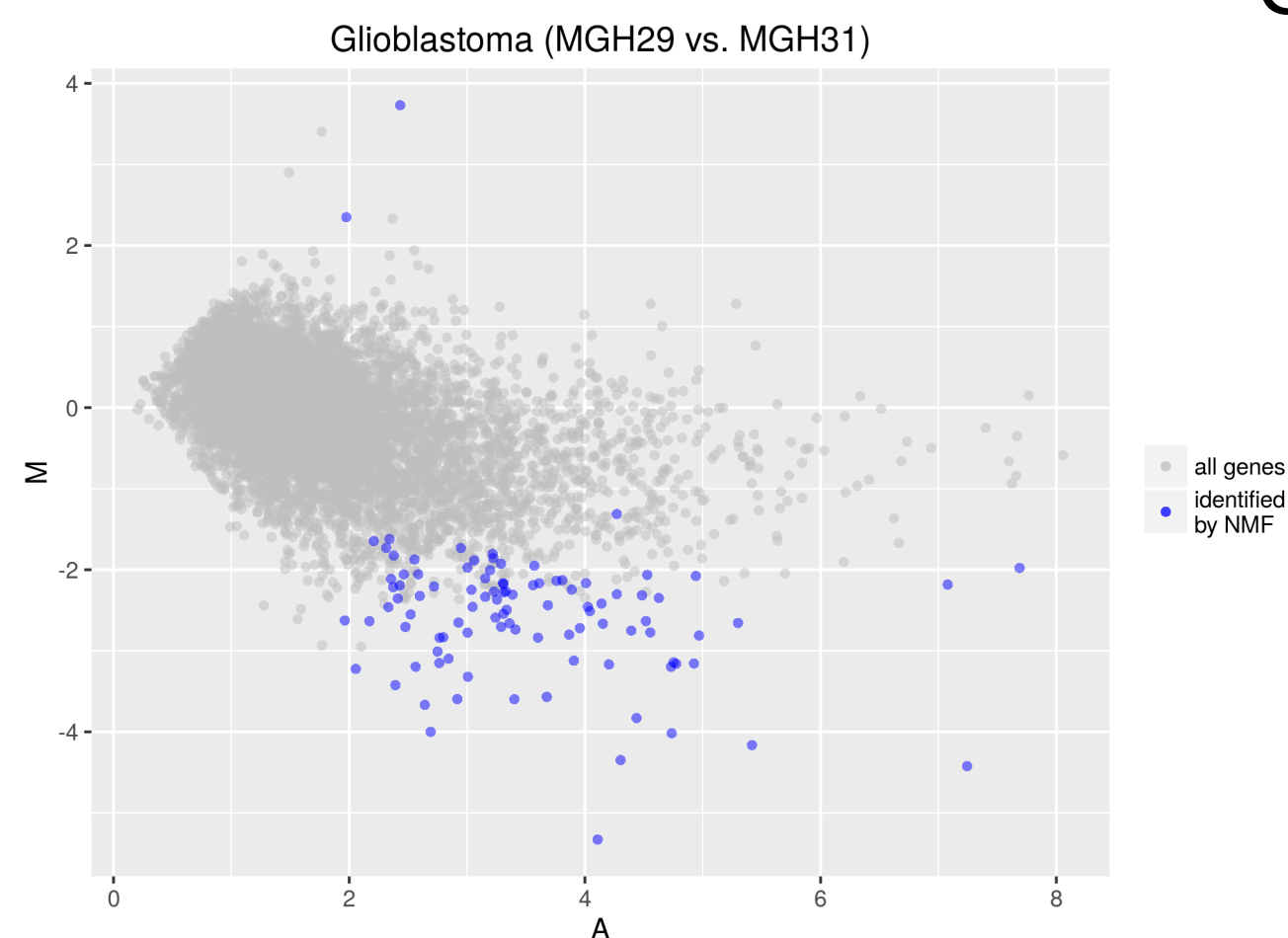

C

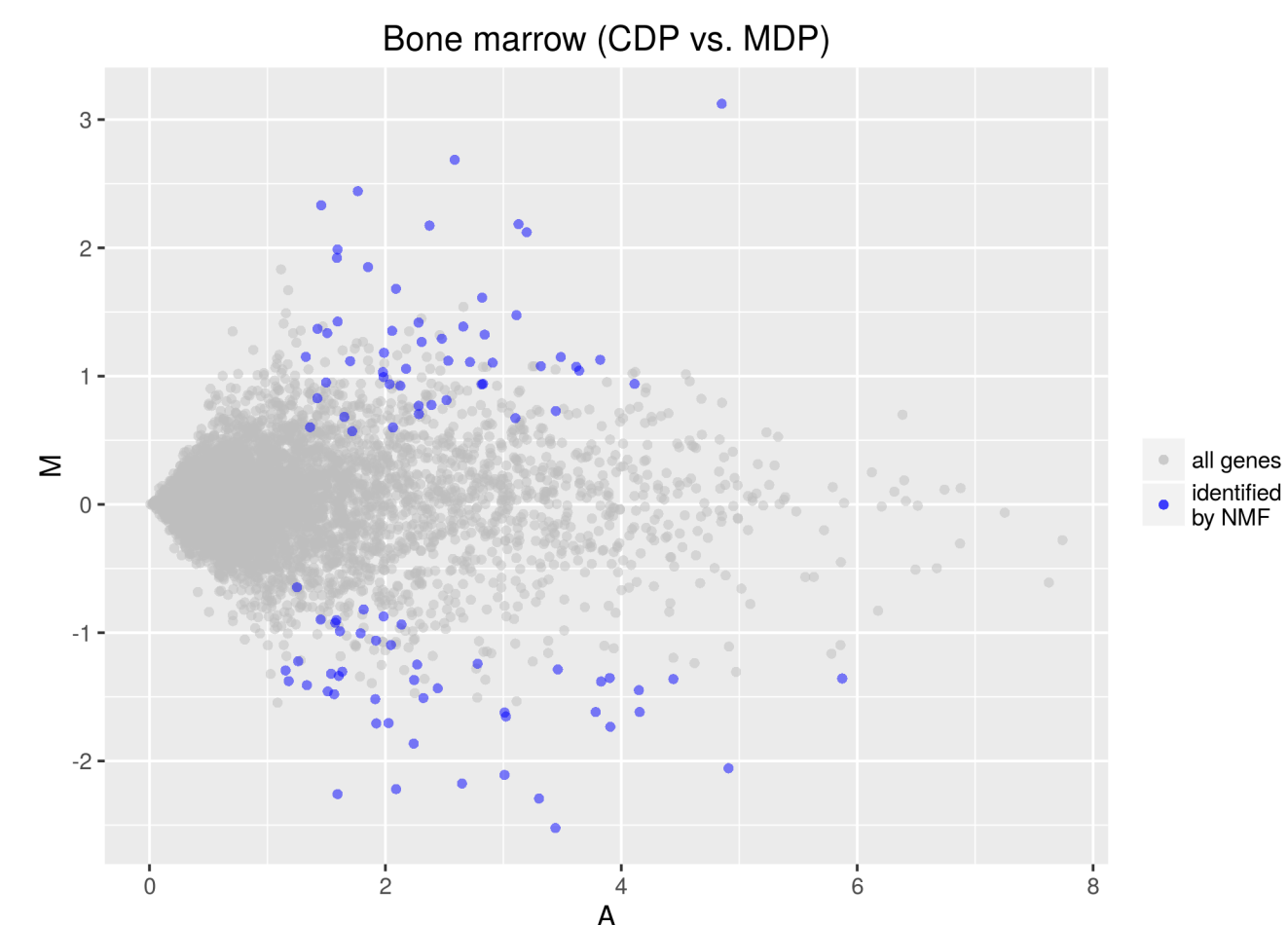

D

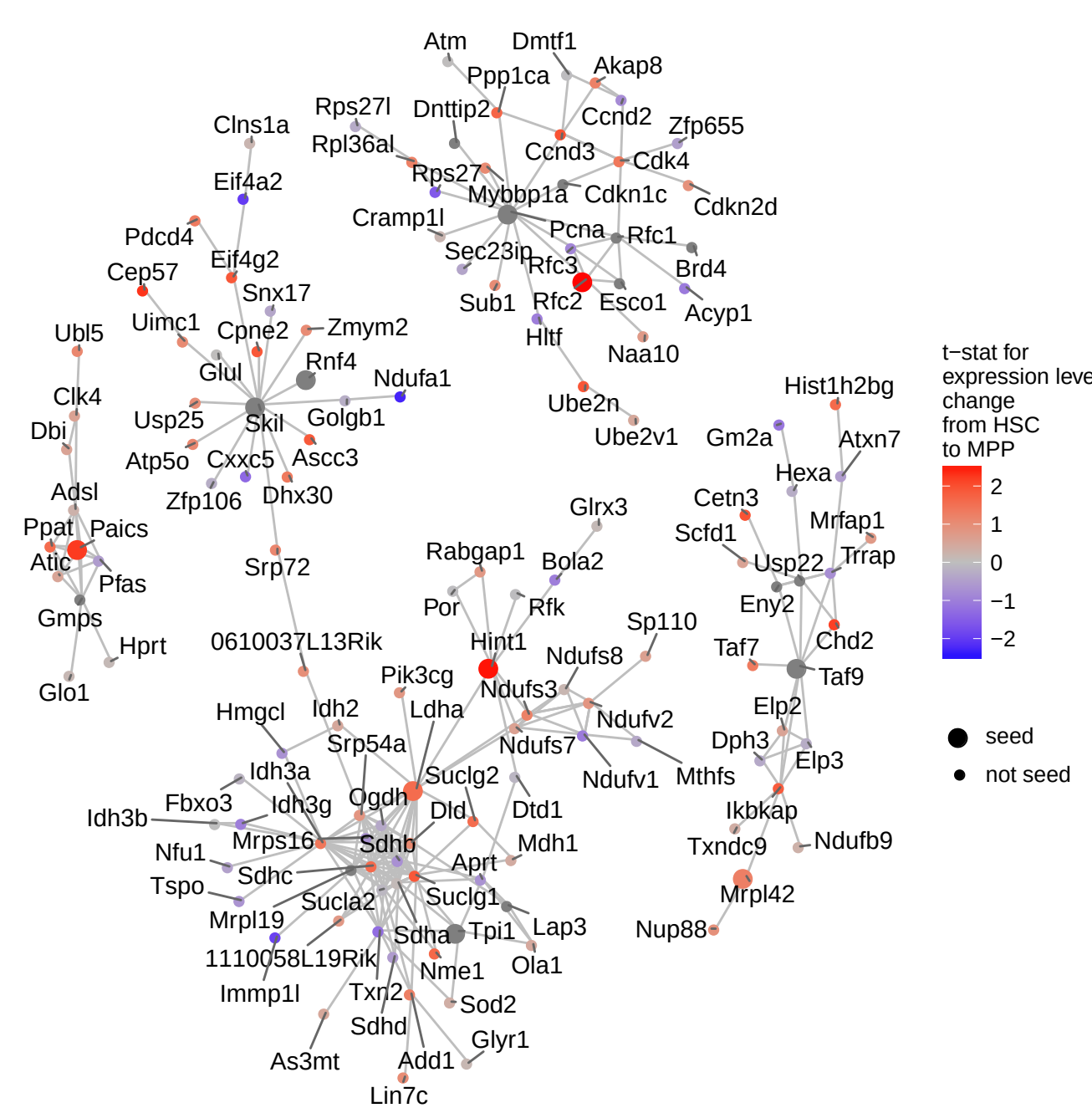

E

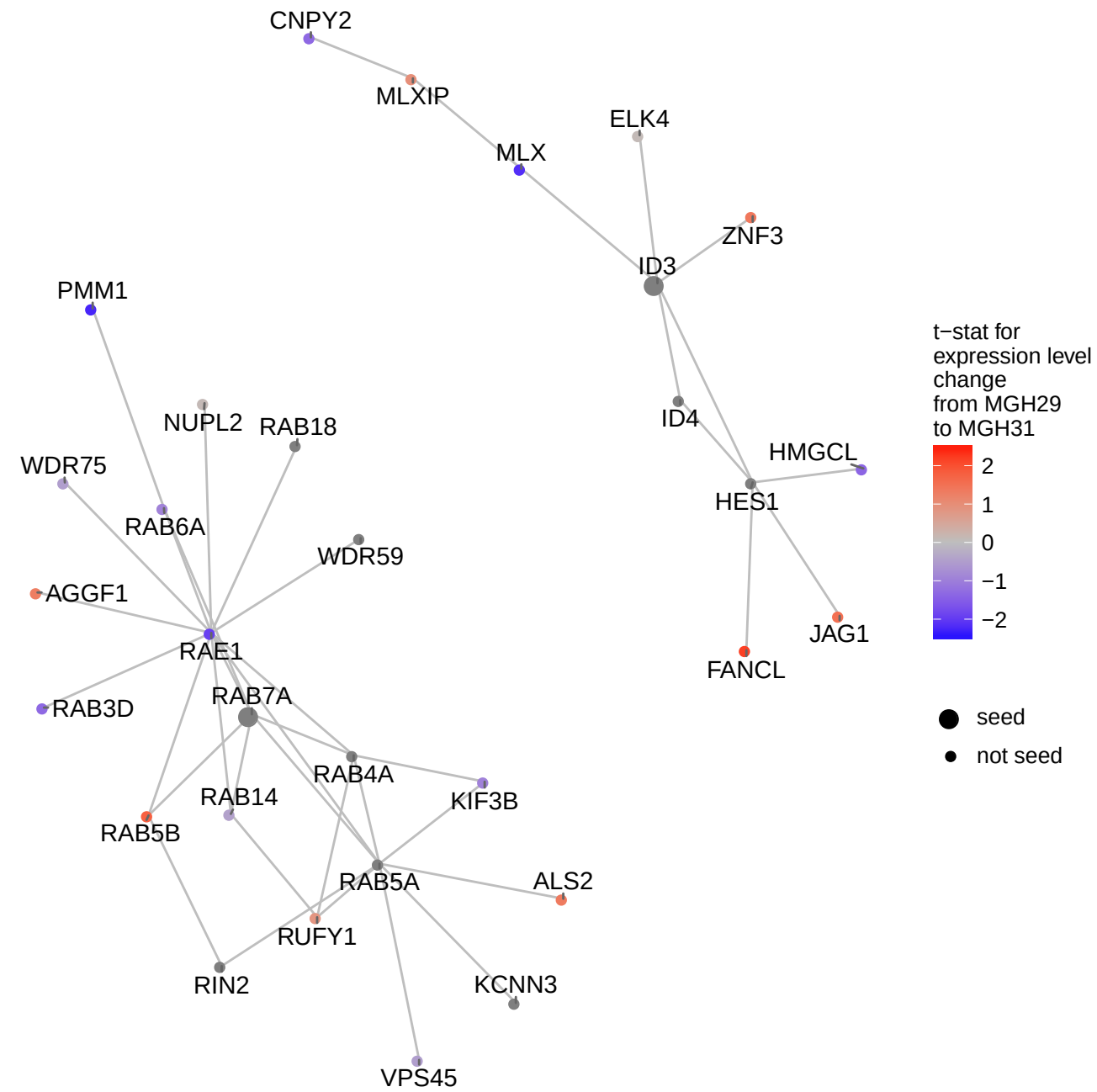

F

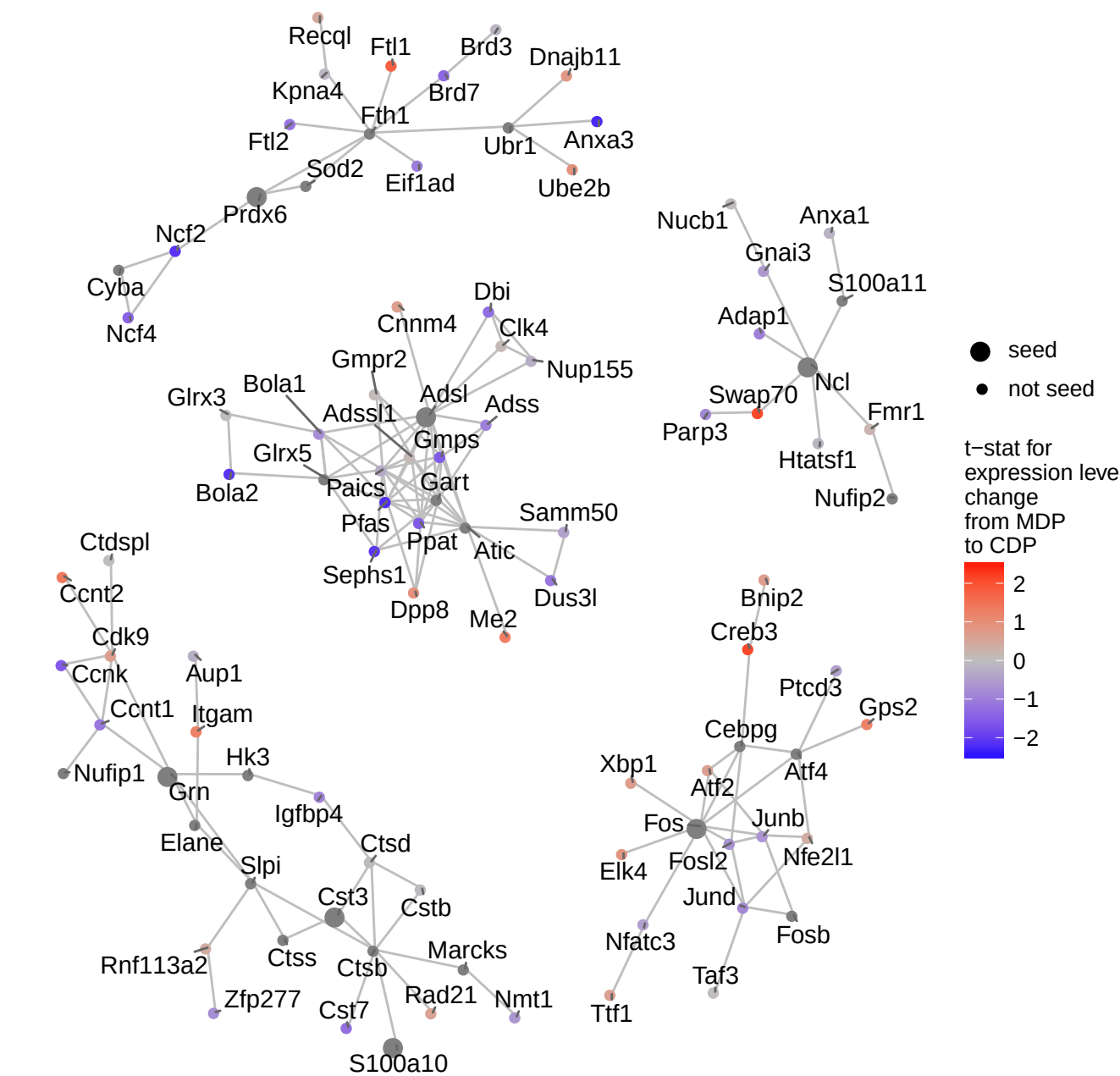

Supplement: Figure S4 — (A–C) MA-plot highlighting the important genes identified by NMF, on three other datasets: (A) HSC vs. MPP1 (B) Glioblastoma MGH29 vs MGH31 (C) Bone marrow dendritic cells CDP vs. MDP. (D–F) Corresponding top PPI network modules discovered by Spinglass algorithm, using genes identified in (A–C) respectively. [file peerj-05-2888-s004.pdf]

Comparing the CV<sup>2</sup> distribution of the genes selected by each method

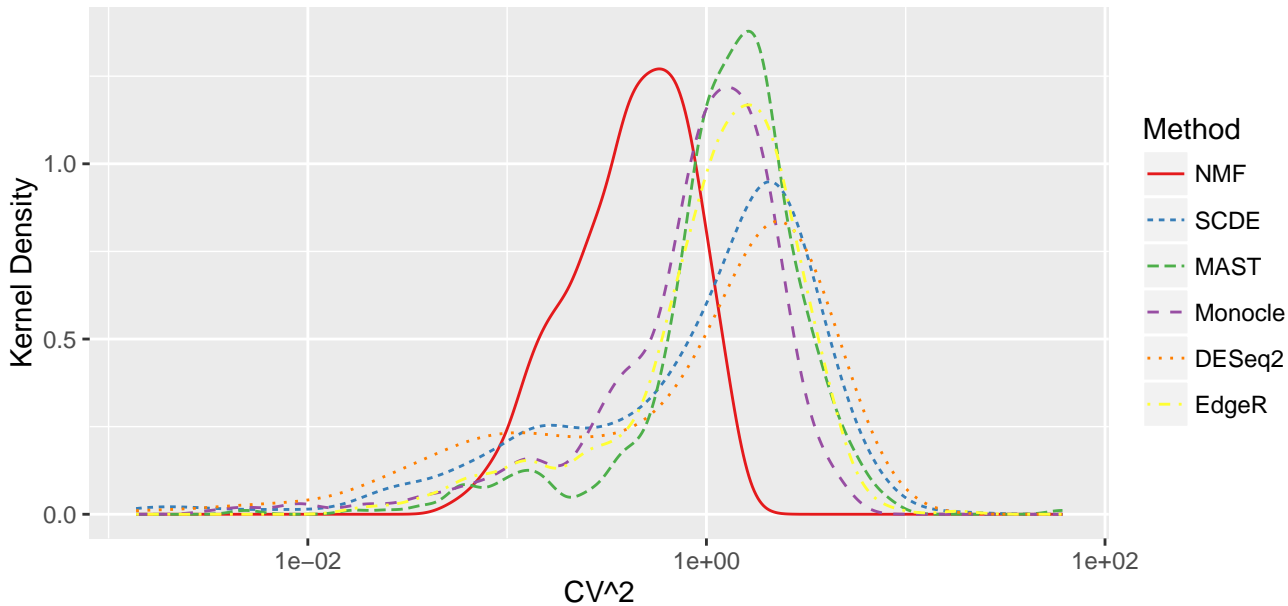

Supplement: Figure S5 [file peerj-05-2888-s005.pdf]

# Comparison in 71 leave-one-sample-out runs

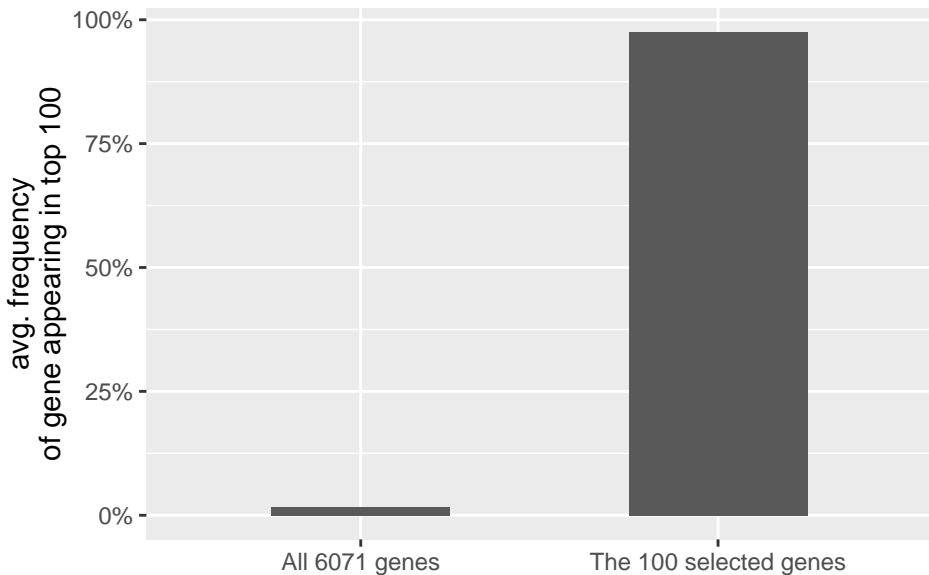

Supplement: Figure S6 — Leave-one-out runs are performed and the selected top 500 genes have over 95% frequency to be in the top 500 gene list, whereas non-top 500 genes have less than 2% frequency to enter the top 500 gene list. [file peerj-05-2888-s006.pdf]
