# Supplementary material for: Detecting heterogeneity in single-cell RNA-Seq data by non-negative matrix factorization
Source: PeerJ. 2017 Jan 19;5:e2888. doi: 10.7717/peerj.2888 (PMC5251935; doi:10.7717/peerj.2888)
Supplement: Figure S3 [file peerj-05-2888-s003.pdf]

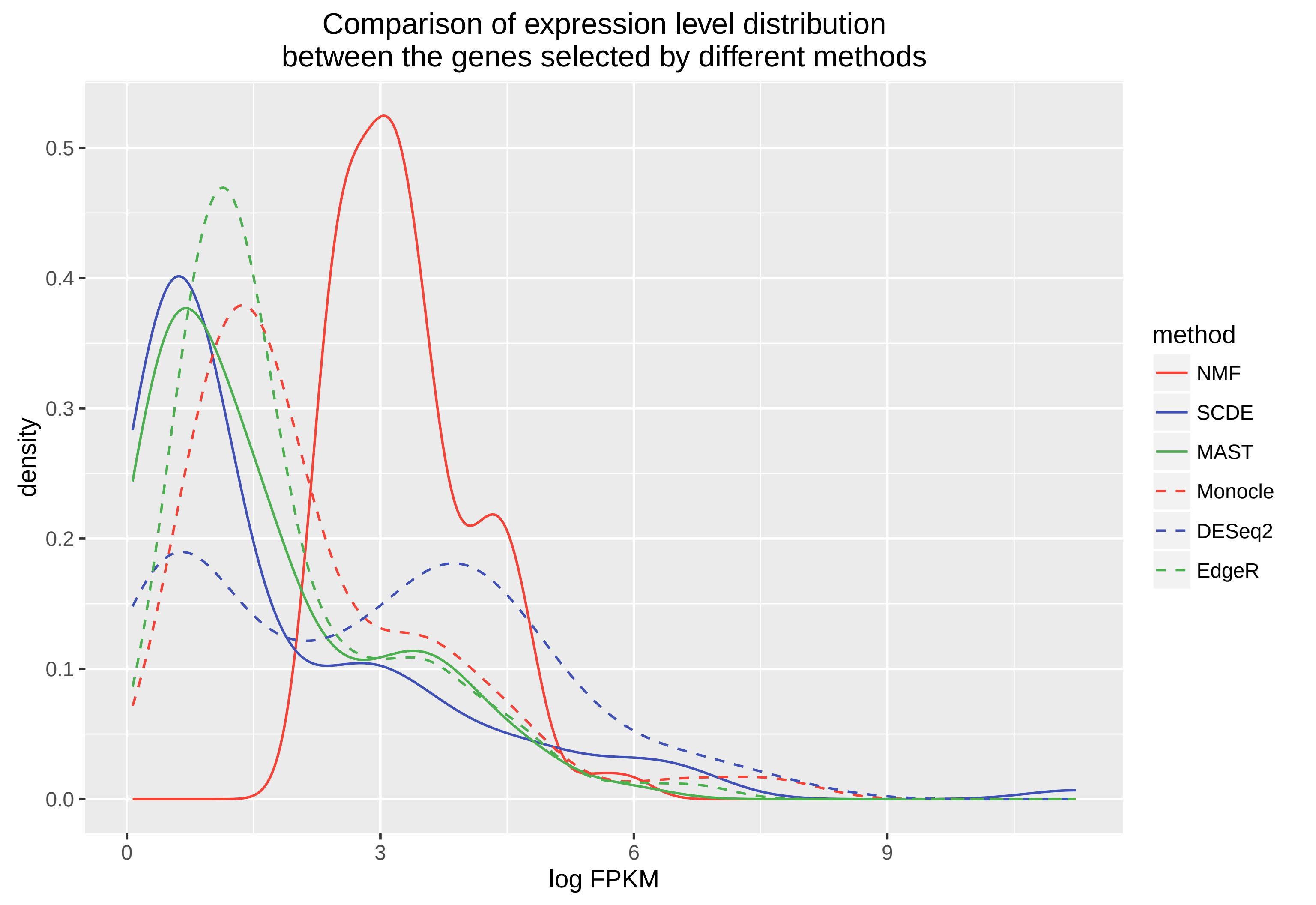

Comparison of expression level distribution
between the genes selected by different methods
density
0.0
0.1
0.2
0.3
0.4
0.5
0
3
6
9
log FPKM
method
NMF
SCDE
MAST
Monocle
DESeq2
EdgeR
